# Supplementary material for: Hemibiotrophic Phytophthora infestans Modulates the Expression of SWEET Genes in Potato (Solanum tuberosum L.)
Source: Plants (Basel). 2023 Sep 29;12(19):3433. doi: 10.3390/plants12193433 (PMC10575152; doi:10.3390/plants12193433)
Supplement: Supplementary file 1 [file plants-12-03433-s001.zip › plants-2586263-supplementary.pdf]

Supplementary Table S1: SWEET proteins with number of amino acids and molecular weight.

Sl. No.	SWEET Name	Protein aa	Molecular Weight
1	StSWEET1	130	14823.5
2	StSWEET2	149	16937.6
3	StSWEET3	219	24470.8
4	StSWEET4	291	32147.2
5	StSWEET5	232	25571.5
6	StSWEET6	233	25761.9
7	StSWEET7	237	26431.4
8	StSWEET8	261	29775.5
9	StSWEET9	280	32153.5
10	StSWEET10	290	32782.3
11	StSWEET11	303	33762.3
12	StSWEET12	296	33086.8
13	StSWEET13	296	33533.2
14	StSWEET14	286	32061.6
15	StSWEET15	253	28119.5
16	StSWEET16	276	30780.5
17	StSWEET17	357	39953
18	StSWEET18	288	32415.1
19	StSWEET19	236	26664.8
20	StSWEET20	253	27791
21	StSWEET21	250	27669
22	StSWEET22	213	24431.5
23	StSWEET23	250	27967.5
24	StSWEET24	200	22166.2
25	StSWEET25	294	33207.1
26	StSWEET26	312	35193.5
27	StSWEET27	256	28236.5
28	StSWEET28	238	26672.8
29	StSWEET29	287	32750.1
30	StSWEET30	289	33158.6
31	StSWEET31	276	31290.5
32	StSWEET32	128	14442.9

33	StSWEET33	230	26330.8
34	StSWEET34	228	25698.8
35	StSWEET35	287	32627.5
36	StSWEET36	133	15062.2
37	StSWEET37	226	25321.5

Supplementary Table S2: SWEET proteins having 3 to 7 TMDs.

Sl. No.	SWEET Name	TMs
1	StSWEET1	3
2	StSWEET2	3
3	StSWEET3	5
4	StSWEET4	7
5	StSWEET5	7
6	StSWEET6	7
7	StSWEET7	7
8	StSWEET8	7
9	StSWEET9	6
10	StSWEET10	7
11	StSWEET11	7
12	StSWEET12	7
13	StSWEET13	7
14	StSWEET14	7
15	StSWEET15	6
16	StSWEET16	7
17	StSWEET17	7
18	StSWEET18	6
19	StSWEET19	7
20	StSWEET20	7
21	StSWEET21	7
22	StSWEET22	6
23	StSWEET23	7
24	StSWEET24	4
25	StSWEET25	7
26	StSWEET26	6
27	StSWEET27	7
28	StSWEET28	7
29	StSWEET29	7

30	StSWEET30	7
31	StSWEET31	7
32	StSWEET32	3
33	StSWEET33	6
34	StSWEET34	6
35	StSWEET35	7
36	StSWEET36	3
37	StSWEET37	6

Supplementary Table S3. Stability index of SWEET protein of potato

SWEET Name	R-	R+	Instability index	Stable/Unstable	Aliphatic index
StSWEET2	9	11	33.56	stable	92.75
StSWEET4	14	30	35.12	stable	111.27
StSWEET6	9	17	34.41	stable	114.59
StSWEET9	25	33	36.26	stable	116.15
StSWEET11	16	27	31.42	stable	122.21
StSWEET12	15	26	37.24	stable	125.37
StSWEET13	20	29	36.11	stable	119.12
StSWEET14	15	26	36.77	stable	128.74
StSWEET15	14	23	34.77	stable	115.85
StSWEET16	16	22	32.91	stable	113.26
StSWEET17	30	35	32.46	stable	109.94
StSWEET18	18	29	39.09	stable	123.12
StSWEET19	13	20	31.28	stable	113.94
StSWEET20	11	22	31.64	stable	113.28
StSWEET21	15	23	29.49	stable	112.28
StSWEET22	14	21	37.04	stable	118.08
StSWEET23	17	24	35.19	stable	123.52
StSWEET24	15	12	27.57	stable	129
StSWEET25	13	26	36.56	stable	103.78
StSWEET28	17	19	26.11	stable	120.76
StSWEET29	20	26	39.67	stable	122.51
StSWEET31	15	26	37.23	stable	132.32
StSWEET32	8	8	34.93	stable	98.2
StSWEET33	10	23	38.92	stable	117
StSWEET34	11	23	36.26	stable	114.52
StSWEET35	24	27	39.97	stable	112.09

StSWEET36	6	9	30.91	stable	133.31
StSWEET37	8	20	36.43	stable	123.72
StSWEET1	7	10	53.01	Unstable	114.62
StSWEET3	13	15	45.69	Unstable	118.31
StSWEET5	8	13	43.71	Unstable	125.17
StSWEET7	8	16	43.86	Unstable	116.41
StSWEET8	20	29	42.19	Unstable	110.08
StSWEET10	28	35	45.26	Unstable	127.95
StSWEET26	19	26	45.34	Unstable	117.76
StSWEET27	11	18	41.55	Unstable	111.6
StSWEET30	22	24	48.9	Unstable	123.32

Supplementary Table S4. RPKM values of SWEET genes

SWEET Nam	Desiree (LB susceptible)	Sarpo mira (Resistant)	SW-92-1015 (Resistant)
StSWEET12	162.47	153.96	136.99
StSWEET13	47.96	35.22	33.18
StSWEET14	111.82	98.42	86.87
StSWEET18	1044.80	1255.36	1089.07
StSWEET27	52.72	36.62	31.59
StSWEET32	10.99	9.01	8.20

Supplementary Table S5. Lesion area (Cm^2) generated by hypersensitive response of *P. infestans* on leaves of K. Bahar

	Lesion area (Cm^2)		
Dpi	R1	R2	R3
Untreated	0	0	0
1	0	0	0
2	0.466	0.39	0.392
3	2.7	1.88	1.38
4	8.552	5.668	4.558
5	15.25	10.95	9.2

Supplementary Table S6. Primer sequences used for qRT-PCR

Sr. No	Gene Name	Primer sequence	Remarks
1	StSWEET10F	TCTATTCAAAGGAGCCGTTCG	
2	StSWEET10R	GCCTAATGGTGCAACGAATAC	
3	StSWEET12F	GGCTGGTATTTCTAATCACTGGG	
4	StSWEET12R	GGAGAAAGGAACACAATGAACG	
5	StSWEET13F	GCAGATCACTGGGCTTTTG	
6	StSWEET13R	TGGCAGTGGAGAAAGGAATAC	
7	StSWEET14F	AGAAAGCCAGGGTCAATACTG	
8	StSWEET14R	CACATAATGAGAAAATAAGGCAAATCC	
9	StSWEET18F	AATTTCTATTCAAAGGAGTCGTTCG	
10	StSWEET18R	TCCACACTCTTGGTCTTGATG	
11	StSWEET27F	GCGGTTATTTCAGTGCTTGC	
12	StSWEET27R	AACGATGGAGAATATGGTAGCAG	
13	StSWEET29F	CTTTGCATTTGGTGTCCTTGG	
14	StSWEET29R	GCACTGAATAAAGCAACCACG	
15	StSWEET31F	CGTTTGTAGCACCATTAGGC	
16	StSWEET31R	AGATGAACCACAGAACAGCAC	
17	StSWEET32F	ATATACGGGCTCTTGAACTGC	
18	StSWEET32R	GCACCAACTGAATTAACCGATG	
19	FPqSNE1	AGGAGAAGGAGGAGAAGAAGAA	*P. infestans* gene primer
20	RPqSNE1	CCGTCATCCTGCTTAGACTTG	*P. infestans* gene primer
21	FPNPP1	TCCTTCAACTCTTCGCATCG	*P. infestans* gene primer
22	RPNPP1	CTCTGTTGTAGTGGTGGTTGT	*P. infestans* gene primer
23	ACTAF	GAATTCGTAACGAGCGTTTCCGTACC	Control
24	ACTAR	GGATCCGCAATG CAGGCATAAACAAG	Control
